# Attitudinal Trends and Misconceptions of Laser Hair Removal Among the Dermatology Patients: A Cross‐Sectional Study

**DOI:** 10.1111/jocd.70332

**Published:** 2025-07-01

**Authors:** Esra Agaoglu, Hilal Kaya Erdogan, Ilayda Kacer, Sibel Sert, Selma Metintas

**Affiliations:** ^1^ Department of Dermatology Faculty of Medicine, Eskisehir Osmangazi University Eskisehir Turkey; ^2^ Department of Public Health Faculty of Medicine, Eskisehir Osmangazi University Eskisehir Turkey

**Keywords:** attitude, hair removal, laser treatment, misconception

## Abstract

**Background:**

Laser hair removal (LHR) is one of the most widely applied cosmetic procedures for the management of unwanted hair. However, some misconceptions or concerns may refrain people from LHR. The aim of this study is to evaluate the attitudes and misconceptions of LHR practices in dermatology patients.

**Methods:**

A cross‐sectional study was conducted on 1002 patients attending the dermatology clinic. They were asked to complete a survey about their LHR history and attitudes, including a questionnaire regarding LHR knowledge and the Chew Health Literacy Scale. A multivariate logistic regression model was used to determine the factors related to having LHR.

**Results:**

Three hundred thirty‐five (33.4%) of the participants had used LHR and among these, 45.1% were treated with Alexandrite laser. Axilla (24.9%) was the most common site of application of LHR. The most frequently reported side effect was transient redness (14.0%). The median score of the LHR knowledge questionnaire and the Chew Health Literacy Scale were higher among those who had used LHR (*p* < 0.001). Participants who were 25–34 years of age, women, university graduates, high income levels, low levels of concern about LHR, high knowledge levels about LHR, and adequate health literacy were independently associated with favoring the application of LHR procedures.

**Conclusions:**

In addition to socioeconomic status, adequate levels of knowledge and health literacy have substantial roles in consideration of laser epilation. Dermatologists should provide sufficient information for the population and prevent the spread of misconceptions about LHR in order to reduce the psychosocial effects of excessive hair growth.

## 1 Introduction

Unwanted hair is a common cosmetic problem in both sexes and ranges from nearly invisible to excessive growth. Although excessive body or facial hair may be associated with medications and underlying disorders such as polycystic ovary syndrome, Cushing syndrome, genodermatoses, or androgen‐secreting tumors, most cases are biologically normal [1, 2]. It is also well known that the presence of excessive hair may affect psychological health and personal relationships, leading to emotional stress, decreased quality of life, social isolation, and depression [3, 4].

The traditional management of unwanted facial and body hair through shaving, waxing, plucking, and chemical depilatories provides temporary solutions for excessive hair growth. Although these methods are less expensive, they may result in certain side effects including pain, swelling, blistering, crusting, as well as pigmentary changes. Additionally, they can be inconvenient and may have variable efficacy [5, 6].

Today, lasers and non‐laser light sources which are considered the most effective methods for long‐term removal of unwanted hair have emerged as the most widely applied cosmetic procedures worldwide. The mechanism of action for laser hair removal (LHR) is based on selective photothermolysis, which involves causing thermal injury to the hair follicle without damaging the surrounding skin. Laser energy targets the melanin in the hair follicles, transforms it into heat, which causes damage to the follicles. Different wavelengths are used in various laser types, including the diode (810 nm) laser, intense pulsed light (IPL) (590–1200 nm) laser, Alexandrite (755 nm) laser, and Neodymium‐doped Yttrium aluminum garnet (Nd:YAG) (1064 nm) laser [5, 7, 8]. There are also numerous low‐energy home IPL devices available today that provide long‐term removal of unwanted hair with personal comfort [9].

Although LHR is associated with a low incidence of side effects that are generally well‐tolerated [10], there may be some misconceptions or concerns that still prevail among the patients, leading to prejudice toward LHR. There are a limited number of studies in the literature addressing the negative opinions and misconceptions about LHR [11, 12, 13], no such study has been reported from our country. The aim of this study is to evaluate the extent of attitudes and misconceptions toward LHR among dermatology patients in our tertiary clinic.

## 2 Materials‐Methods

### 2.1 Study Design and Sample

This cross‐sectional study was performed on patients who are randomly attending our tertiary clinic (Eskisehir/Türkiye) between October 2023 and January 2024. The local ethics committee approved the study protocol (decision no: 2023/274). Informed consent was taken from the participants before being included in the study. Participants aged between 18 and 80 years old were asked to fill out the questionnaire form. The sample size was calculated; a minimum of 964 people was calculated with an alpha error of 5%, the study's power (1‐Beta) of 80%, and an effect size of 10%. Considering possible losses in the study group, the sample size was increased by 5%, and the study group consisted of 1002 participants.

### 2.2 Data Collection

The first part of the survey sociodemographic characteristics, including age, sex, educational level, and monthly income levels, was evaluated. According to their perception, the participants' income level was evaluated as low, medium, or high. Additionally, the presence of LHR experience, body parts that were used for LHR, and the presence of any adverse effects during LHR sessions were asked. The skin types of the participants were recorded by a dermatologist according to the Fitzpatrick classification [14].

The second part of the survey consisted of participants' concerns; knowledge levels related to LHR and health literacy were evaluated. Participants were asked whether they had any concern about LHR, and the anxiety level regarding LHR was evaluated with the Visual Analog Scale (VAS). VAS is a single‐item measurement that is commonly used in healthcare settings to measure anxiety related to medical treatments. The scale value is between 0 and 10 [15]. Regarding the knowledge levels and concerns about LHR, we used a self‐administered questionnaire consisting of 11 statements in the study, and the questionnaire was created mainly from the questions that were frequently asked by our patients about LHR. The participants were asked to rate the statements in the questionnaire on a three‐point Likert‐type scale format with the following response options: agree, indecisive, and disagree. The total scores ranged from 0 to 22. The higher the total score obtained from the questionnaire was referred to as adequate knowledge about LHR. The Cronbach alpha coefficient of the questionnaire was found to be 0.858.

The Turkish version of the Chew Health Literacy Scale was used in the survey [16] and questions were answered on a 5‐point Likert scale ranging from 0 (always) to 4 (never). Based on the scale, the mean scores of the questions were utilized. Patients with a mean score of 2.5 or lower were categorized as having an inadequate level of health literacy, while those scoring above 2.5 were categorized as having a sufficient level of health literacy. In this study, the Cronbach's alpha coefficient of the Chew Health Literacy Scale was found to be 0.692.

### 2.3 Statistical Analysis

Statistical analysis performed using SPSS Statistics v15 (SPSS Inc., Chicago, IL, USA) software program. Descriptive statistics were utilized to evaluate the data, including numbers, percentages, means, standard deviations, medians, minimum, and maximum values. Participants' concern level, level of knowledge about LHR, and health literacy data were tested for normal distribution. These data were not normally distributed, so the Mann–Whitney *U* test was used for comparisons between groups. Chi‐square (*χ* ^2^) analysis was used to compare frequencies. Since 25% of the expected values were less than 5, Fisher's *χ* ^2^ test was used in 2 × 2 tables, and the Bootstrap method was used in 2 × m tables. Logistic regression analysis was used to calculate crude and adjusted odds ratios (OR). The dependent variable was defined as the status of using LHR, and independent variables affecting this status were evaluated based on the related variables. Multiple logistic regression models were applied in the second step to identify independent risk factors for using LHR. Variables with *p*‐values greater than 0.10 were not included in the stepwise model.

## 3 Results

### 3.1 Sociodemographics and Clinic Data

Of the 1002 participants, 68.5% were women and 31.5% were men. Six hundred eighty‐six (68.5%) of the participants were female and 316 (31.5%) were male. The mean age of the patients was 36.67 ± 13.98 years (range = 18–79). About half of the patients were university graduates or higher, and 46.4% of the patients did not have an income‐generating job. Most participants (76.0%) described their income as moderate. According to the Fitzpatrick classification, the most common skin types of the patients were type III (47.5%) and type II (24.7%), respectively.

### 3.2 Practices of LHR

Three hundred thirty‐five (33.4%) of the participants had previous experience of LHR.

**FIGURE 1:**
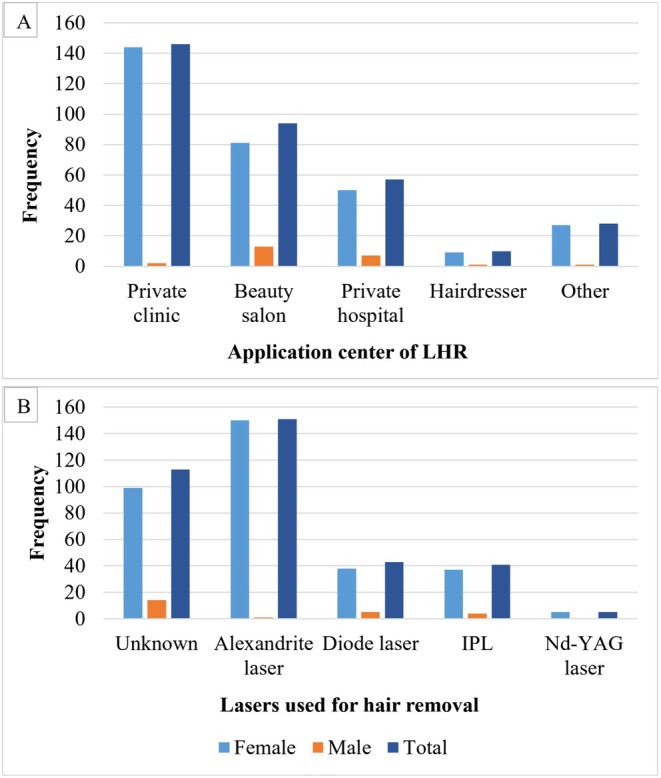
(A) Distribution of application centers for laser hair removal (LHR) by gender. (B) Distribution of laser types used for hair removal by gender.

**FIGURE 2:**
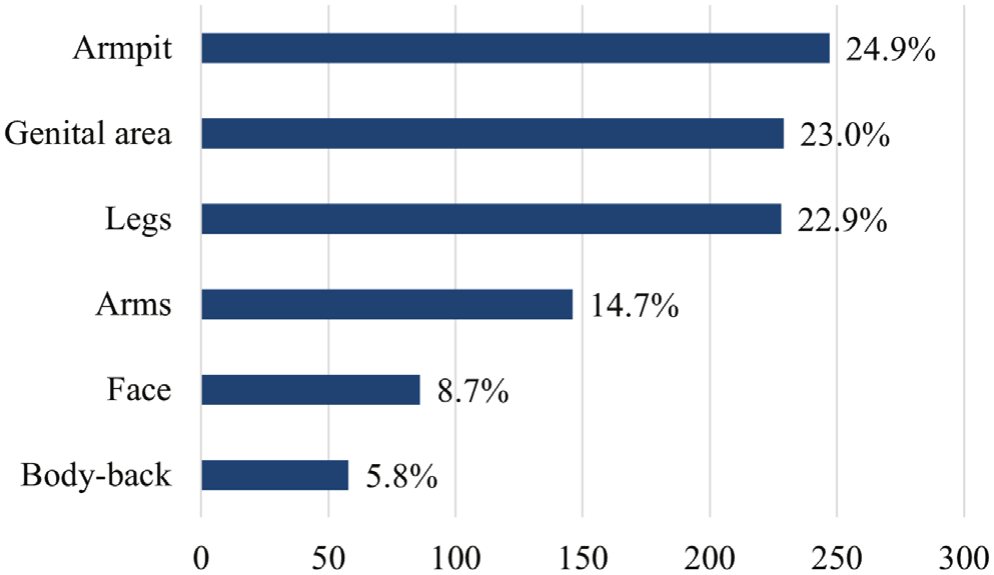
Body parts treated with LHR.

**FIGURE 3:**
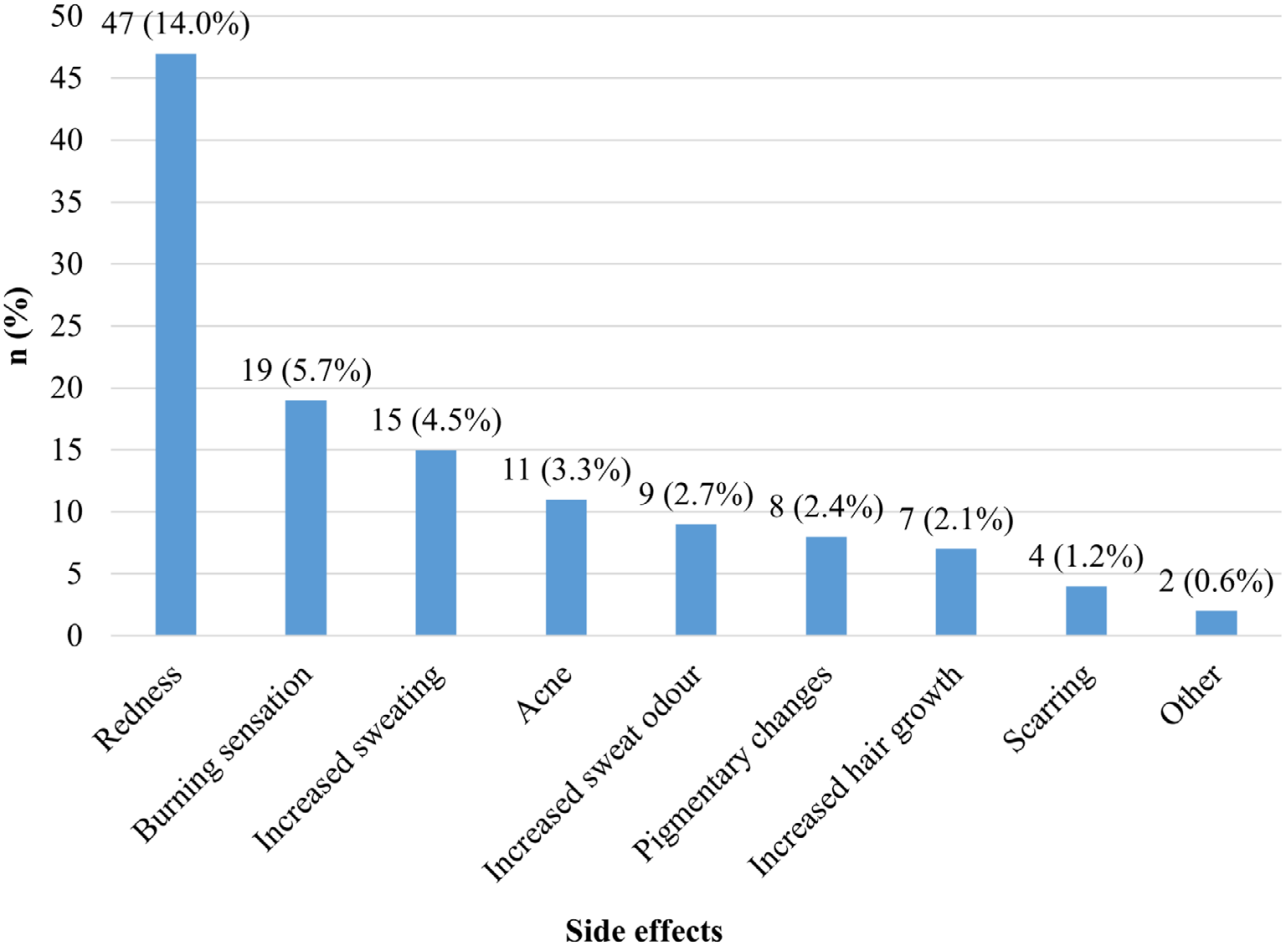
Side effects related to LHR.

Of the patients who had LHR, 311 (92.8%) of them were women. No statistically significant difference was found between the age groups, educational level, marital status, place of residence, employment status, monthly income, severity of concern, and health literacy levels of the female and male participants who had LHR (*p* > 0.05) (Table 1). Of the female participants, 144 (46.3%) had LHR in a private clinic, 81 (26.9%) in a beauty salon, and 50 (16.0%) in a private hospital. Of the male participants, 13 (54.1%) had laser treatment in a beauty salon, 7 (29.1%) in a private hospital, and 2 (8.3%) in a private clinic. One hundred fifty (48.2%) female participants stated that Alexandrite laser was used for LHR, while 14 (58.3%) male participants did not know which laser device was used (Figure 1). The most common areas of LHR application were the axillary region (24.9%), the genital region (23.0%) and the legs (22.9%) (Figure 2). Side effects were reported by 85 (25.4%) of the participants. No significant difference was found in terms of side effects between male and female participants who had LHR (*p* = 0.156). The most commonly reported side effects related to LHR were transient redness, burning sensation, and axillary hyperhidrosis (14.0%, 5.7%, 4.5%) respectively. Figure 3 summarizes all reported side effects.

**TABLE 1:** Comparison of the characteristics of male and female participants who had LHR.

Variables	LHR history (%)		
	Women *n* = 331 (%)	Men *n* = 24 (%)	*p*
Age groups			
18–24	88 (28.3)	5 (20.8)	0.1101^b^
25–34	77 (24.8)	11 (45.8)	
35–44	81 (26.0)	7 (29.2)	
45–54	49 (15.8)	1 (4.2)	
≥ 55	16 (5.1)		
Education			
Primary school	36 (11.6)	—	0.073^b^
High school	58 (18.6)	8 (33.3)	
University	217 (69.8)	16 (66.7)	
Marital status			
Single	150 (48.2)	15 (62.5)	0.178^b^
Married	161 (51.8)	9 (37.5)	
Residence			
Rural	41 (13.2)	1 (4.2)	0.334^a^
Urban	270 (86.8)	23 (95.8)	
Occupation			
Unemployed	159 (51.1)	7 (29.2)	0.063^b^
Employed	152 (48.9)	17 (70.8)	
Monthly income			
Low	31 (10.0)	^—^	0.167b
Middle	237 (76.2)	22 (91.7)	
High	43 (13.8)	2 (8.3)	
Severity of concern			
High	113 (36.3)	4 (16.7)	0.081^b^
Low	198 (63.7)	20 (83.3)	
Level of health literacy			
Inadequate	74 (23.8)	5 (20.8)	0.742^b^
Adequate	237 (76.2)	19 (79.2)	

^a^ Fisher's Exact test.

^b^ Mann–Whitney *U* test.

### 3.3 Concerns and Knowledge Level Regarding LHR and Health Literacy

The participants' responses to the LHR knowledge questionnaire are presented in Table 2.

**TABLE 2:** The distribution of the participants' answers to the items related to the LHR questionnaire.

Items	Yes *n* (%)	No *n* (%)	Do not know *n* (%)
1. Laser epilation could be used to removal of unwanted hair permanently (T)	359 (35.8)	250 (25.0)	393 (39.2)
2. Laser epilation emits radiation (F)	248 (24.8)	249 (24.9)	505 (50.3)
3. Laser epilation should not be used during pregnancy (T)	522 (52.1)	68 (6.8)	412 (41.1)
4. Laser epilation should not be used during lactation (T)	489 (48.8)	66 (6.6)	447 (44.6)
5. Laser epilation should not be used for children under 12 years of age except in clinically indicated cases (T)	643 (64.2)	42 (4.2)	317 (31.6)
6. Laser epilation may cause skin cancer (F)	206 (20.6)	172 (17.2)	624 (62.3)
7. Laser epilation applied to the genital area may cause infertility (F)	90 (9.0)	340 (33.9)	572 (57.1)
8. Laser epilation applied to the armpit may damage the lymph nodes and cause lymph cancer (F)	258 (25.7)	106 (10.6)	638 (63.7)
9. Laser epilation may cause breast cancer (F)	268 (26.7)	95 (9.5)	639 (63.8)
10. Safety glasses should be used during laser epilation (T)	685 (68.4)	37 (3.7)	280 (27.9)
11. After laser epilation, the application area should be protected from the sun (T)	706 (70.5)	29 (2.9)	267 (26.6)

Abbreviations: F, false; T, true.

The mean score of the questionnaire was 10.52 ± 6.26 (median = 11), and 56.4% of the participants scored above mean score.

The median score of the LHR knowledge questionnaire was 14.0 (0–22) in participants who had used LHR and 10.0 (0–22) in participants who did not have used LHR. The knowledge level among participants was significantly higher among those who had used LHR compared to those who did not use LHR (*p* < 0.001). The median score of female participants who had LHR was 15 (11–18), while the median score of male participants was 11.5 (8–13). The knowledge level of female participants who had LHR was found statistically higher than that of male participants (*p* = 0.03).

According to the LHR knowledge questionnaire, 268 (26.7%) of the participants thought that “Laser epilation may cause breast cancer”, while 258 (25.7%) of the participants reported that “Laser epilation applied to the armpit may damage the lymph nodes and cause lymph cancer”. Additionally, 172 (17.2%) of the participants believed that” Laser epilation emits radiation” (Table 2).

**FIGURE 4:**
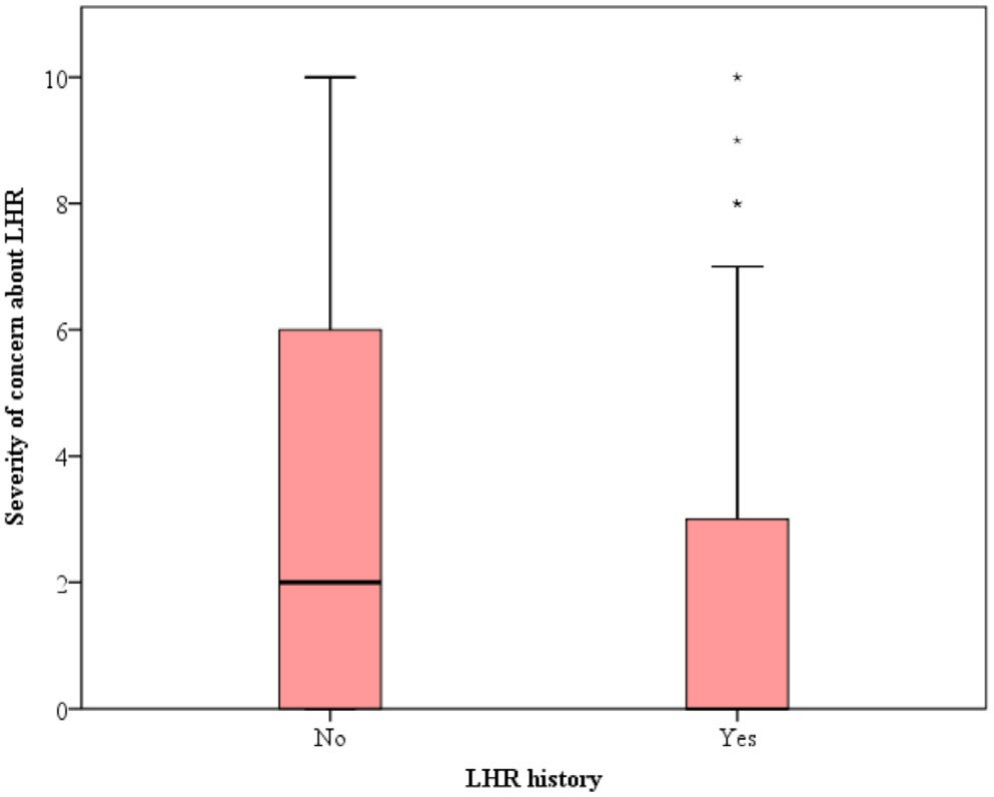
Comparison of the participants' concern severity according to LHR history.

Forty‐five percent of the participants were concerned about LHR, and the mean VAS score of the participants was 2.59 ± 3.20 (median: 0). The VAS score of those who used LHR (1.63 ± 2.53) was statistically significantly lower than that of those who did not use LHR (3.08 ± 3.38) (*p* < 0.001) (Figure 4).

The mean score of the Chew Health Literacy Scale of the participants was 3.03 ± 0.65 (median = 3) and 73% of the participants have an adequate level of health literacy. The median Chew Health Literacy Scale score of the participants who had used LHR was 3.25 (1.25–4) and 3 (1.0–4.0) in patients who did not use LHR. The participants who had used LHR had a more adequate level of health literacy than participants who did not use LHR (*p* < 0.001).

### 3.4 Parameters Associated With Using LHR

Logistic regression analysis was performed to determine the independent parameters of the participants' affecting LHR procedure. Variables associated with the decision to use LHR showed age‐related changes in univariate analysis. Participants in the 25–34 age group had used LHR more than participants in the 18–24 age group and those aged above 55. Additionally, it was found that individuals who are women, university graduates, married individuals, those living in urban areas, those with moderate to high income levels, those with low‐level concerns about LHR, and those with higher knowledge levels were more likely to use LHR. However, in the multivariate logistic regression analysis, marital status, income status, and location of residence lost significance after adjusting for other characteristics. Conversely, those with adequate health literacy were found to have higher odds of using LHR. The univariate and multivariate logistic regression analysis results of the related factors are given in Table 3.

**TABLE 3:** Variables regarding the LHR history of participants (univariate and multivariate logistic regression analysis).

Variables	LHR history (%)	Unadjusted OR (95% Cl)	*p*	Adjusted OR (95% Cl)	*p*
Age groups					
18–24	34.4	1.0		1.0	
25–34	43.3	1.46 (1.00–2.12)	**0.049** *	1.86 (1.08–3.20)	**0.025** *
35–44	37.6	1.15 (0.80–1.65)	0.461	1.84 (1.00–3.40)	0.051
45–54	28.7	0.77 (0.51–1.16)	0.209	1.10 (0.57–2.11)	0.781
≥ 55	13.2	0.29 (0.16–0.52)	**< 0.001** *	0.54 (0.25–1.18)	0.121
Sex					
Men	7.6	1.0		1.0	
Women	45.3	10.09 (6.48–15.71)	**< 0.001** *	12.59 (7.62–20.81)	**< 0.001** *
Education					
Primary school	18.1	1.0		1.0	
High school	25.7	1.57 (0.99–2.47)	0.055	1.72 (0.96–3.08)	0.070
University	42.7	3.37 (2.26–5.02)	**< 0.001** *	2.83 (1.59–5.01)	**< 0.001** *
Marital status					
Single	37.2	1.0		1.0	
Married	30.4	0.74 (0.57–0.96)	**0.023** *	0.70 (0.44–1.09)	0.116
Residence					
Rural	23.6	1.0		1.0	
Urban	35.6	1.79 (1.23–2.60)	**0.002** *	1.40 (0.90–2.19)	0.138
Occupation					
Unemployed	30.9	1.0		1.0	
Employed	36.3	1.28 (0.98–1.66)	0.069	1.29 (0.88–1.89)	0.193
Monthly income					
Low	20.5	1.0		1.0	
Middle	33.6	1.96 (1.28–2.99)	**0.002** *	1.24 (0.73–2.12)	0.423
High	56.2	4.98 (2.76–9.00)	**< 0.001** *	2.60 (1.24–5.46)	**0.012** *
Fitzpatrick skin type					
V	36.8	1.0			
IV	31.3	0.78 (0.30–2.06)	0.615		
III	35.1	0.93 (0.36–2.39)	0.875		
II	32.0	0.81 (0.31–2.13)	0.663		
I	35.0	0.92 (0.25–3.42)	0.905		
Severity of concern					
High	25.5	1.0		1.0	
Low	40.1	1.95 (1.49–2.56)	**< 0.001** *	2.90 (2.08–4.05)	**< 0.001** *
Level of health literacy					
Inadequate	29.2	1.0		1.0	
Adequate	35.0	1.31 (0.97–1.77)	0.081	1.60 (1.08–2.37)	**0.019**
Adequate knowledge level	—	1.16 (1.13–1.19)	**< 0.001** *	1.10 (1.06–1.13)	**< 0.001** *

* Statistically significant values.

## 4 Discussion

Unwanted body hair is a significant distressful condition and may affect the daily lives of individuals. Patients with excessive hair may experience feelings of embarrassment and lower self‐confidence, leading to a considerable psychological burden. It is documented that patients with excessive hair experience a low quality of life comparable with chronic skin diseases such as psoriasis and eczema [2, 3, 17, 18].

Since societal acceptability is an important motivator in interpersonal communications, there is an increasing request for cosmetic procedures in society. Today, LHR treatments are considered one of the most effective procedures for reducing excessive hair growth. Laser epilation delays hair growth, removes large areas of hair with minimal discomfort and with few complications. It is also documented that quality of life and measures of anxiety and depression have been improved with LHR [3, 19]. Although there are considerable variations related to skin or hair color, hair thickness, and anatomical region, an approximately 80% reduction in hair growth is achieved in patients with fair skin and dark hair [2, 6, 7, 20]. Additionally, patient satisfaction after LHR remains the best method compared with other hair removal options [5].

In this study, we conducted a cross‐sectional survey regarding the participants' attitudes and perceptions about LHR. Three hundred thirty‐five (33.4%) of the participants had a history of LHR, and 311 (92.8%) of them were women. It appears that women experience more discomfort regarding excessive hair growth than men and are more likely to seek treatment for it. Additionally, when female and male participants who had LHR were compared, no statistically significant difference was found between the sociodemographic characteristics, severity of concern levels regarding LHR, and health literacy levels. However, according to the LHR knowledge questionnaire, female participants' knowledge level was found to be higher than male participants. This situation can be explained by the female predominance in our study, and therefore, they may have more knowledge on LHR treatments.

Similar to the previous studies [13, 21], the most frequently (38.5%) procedural reaction after LHR was transient redness in our study. Actually, acute short‐term reactions after LHR such as transient erythema and edema, which are considered as real manifestations of effective laser photothermolysis, usually last from hours to days and resolve spontaneously or with topical corticosteroids [9].

The axillary hyperhidrosis is another reported adverse effect secondary to LHR. The possible mechanism of hyperhidrosis may be attributed to the thermal heating following laser energy, which may stimulate the nerve fibers that innervate the eccrine glands of the applied area. It is also suggested that laser‐induced hypersecretion is linked to the thermoregulation center of the anterior hypothalamus [22, 23]. Helou et al. reported a prospective study that included 406 female patients who had axillary LHR. The incidence of axillary hyperhidrosis was found to be 11% of the patients following diode and alexandrite laser treatments [24]. However, Aydin et al. reported persistent axillary hyperhidrosis with Nd‐YAG laser, which had continued for 1 year after the treatment [23]. In our study, axillary hyperhidrosis was reported in 12.3% of the participants, and Alexandrite laser was the most commonly used laser in these individuals.

We developed a knowledge questionnaire consisted of 11 items to focus on awareness and misconceptions of the participants regarding LHR. Our study demonstrated that the median score of the LHR knowledge questionnaire was significantly higher among those who had used LHR compared to those who did not used LHR. According to the questionnaire, the most frequently incorrectly marked item was; “Laser epilation could be used for long‐term removal of unwanted hair”. This opinion may be due to the participant's knowledge that laser epilation requires multiple sessions to reach optimal results. Additionally, the best well‐known issues regarding the safety procedures of LHR are protecting the application area from the sun after epilation (70.5%) and the use of protective glasses during laser epilation (68.4%).

In our study approximately half of the participants have concerns about LHR. However, the mean VAS score of those who used LHR was statistically significantly lower than those who did not use LHR. According to our questionnaire the most common misconceptions related to LHR were the concern of breast cancer (26.7%) and lymph cancer (25.7%). These concerns were revealed for the first time in our study and such misconceptions of course may be a reason for abstaining from LHR procedures.

Laser is an acronym for Light Amplification by Stimulated Emission of Radiation and the term “radiation” provoke alarm in the general population. Twenty‐four percent of our participants believed that laser epilation emits radiation and 20.6% of them thought that laser epilation may cause skin cancer. Although there is no evidence that laser treatments may cause skin cancer [25], there is still a concern about this issue in the population. In a study by AlGhamdi & Moussa, they explored the misconceptions about lasers in dermatology patients and revealed that 21.4% of the patients believe that laser treatments can cause skin cancer [11]. Similarly, in a recent study by Al Hatmi et al., approximately 10% of the participants thought that LHR could cause skin cancer [13]. These differences may be explained by the variety of cultural beliefs.

Health literacy is the ability to perform basic reading and decision‐making skills that are necessary in a healthcare environment [26]. It is also defined as the personal characteristics and social resources needed for people to access, understand, and use information when making health‐related decisions [27]. Individuals with a high level of health literacy can better obtain, understand, evaluate, and apply the accurate information necessary for medical decision‐making [28, 29]. Today, with easily accessible information sources such as the internet, an increasing number of people with low health literacy levels use this source to obtain their first‐line health information [30, 31]. Individuals with low health literacy are 1.5 to 3 times more likely to experience a poor outcome after a procedure related to inadequate or misunderstanding of healthcare resources [32]. In our study, when participants were evaluated with the Chew Health Literacy Scale, health literacy levels were found to be higher in those who had LHR compared to those who did not, suggesting that these individuals have enough confidence for applying LHR procedures.

The correlation between LHR procedures and sociodemographic and knowledge levels of the participants was documented with multivariate logistic regression analyses. When the participants' tendencies to use LHR procedures were evaluated, it was found that those who were 25–34 years old, women, university graduates, those with high income levels, those with low concerns about LHR, those with high knowledge levels about LHR, and those with adequate health literacy were more likely to use LHR. Our study revealed that young women with higher education and better financial incomes more frequently opted for LHR. Similar results were also reported in a previous study which was conducted in African‐Americans, as it was found that female subjects, subjects with both higher educational backgrounds and annual incomes have a higher tendency to consider LHR [12]. Additionally, in our study, individuals with high levels of knowledge and health literacy exhibited a more positive attitude toward LHR.

Limitations of this study include that it was conducted only in one city and reflects the patients' misconceptions and knowledge from a single center. Moreover, the cross‐sectional study type limits our results in causality. Studying LHR concerns throughout the country may provide clinical insights into patients' perception of this problem.

## 5 Conclusion

Our findings revealed that socioeconomic status is one of the determinant factors in individuals' preference for LHR. Additionally, individuals with higher levels of knowledge and health literacy exhibited a more positive attitude toward LHR, which emphasizes the importance of education in overcoming prejudices. It should aim to reduce the level of anxiety regarding LHR and improve the level of education and health literacy. Myths and misconceptions related to LHR should be eliminated in order to reduce the psychosocial effects of excessive hair growth. Dermatologists should provide accurate and sufficient information for the population and prevent the widespread misconceptions about LHR.

## Author Contributions

All authors read and accepted the last manuscript. E.A., H.K.E. and S.M. designed the research study. E.A., H.K.E., I.K., S.S., and S.M. performed the research. E.A., H.K.E., I.K., S.S., and S.M. analyzed the data. E.A., H.K.E. and S.M. wrote the paper.

## Ethics Statement

The local ethics committee approved the study protocol (decision no.: 2023/274).

## Conflicts of Interest

The authors declare no conflicts of interest.

## Data Availability

The data that support the findings of this study are available from the corresponding author upon reasonable request.
